# 2288 Neural correlates of face processing in autism spectrum disorder: A quantitative meta-analysis of current literature and future directions

**DOI:** 10.1017/cts.2018.100

**Published:** 2018-11-21

**Authors:** Carla J. Ammons, Mary-Elizabeth Winslett, Rajesh K. Kana

**Affiliations:** 1 University of Alabama, Birmingham, AL, USA; 2 Department of Psychology, University of Alabama, Birmingham, AL, USA

## Abstract

OBJECTIVES/SPECIFIC AIMS: Autism spectrum disorder (ASD) affects 1 in 68 people and includes restricted, repetitive behavior, and social communication deficits. Aspects of face processing (i.e., identity, emotion perception) are impaired in some with ASD. Neuroimaging studies have shown aberrant patterns of brain activation and connectivity of face processing regions. However, small sample sizes and inconsistent results have hindered clinical utility of these findings. The study aims to establish consistent patterns of brain responses to faces in ASD and provide directions for future research. METHODS/STUDY POPULATION: Neuroimaging studies were identified through a multi-database search according to PRISMA guidelines. In total, 23 studies were retained for a sample size of 383 healthy controls and 345 ASD. Peak coordinates were extracted for activation likelihood estimation (ALE) in GingerALE v2.3.6. Follow-up ALE analyses investigated directed Versus undirected gaze, static Versus dynamic, emotional Versus neutral, and familiar Versus unfamiliar faces. RESULTS/ANTICIPATED RESULTS: Faces produced bilateral activation of the fusiform gyrus (FG) in healthy controls (−42 −52 −20; 22 −74 −12, *p*<0.05, FDR) and left FG activation in ASD (−42 −54 −16, *p*<0.05, FDR). Activation in both groups was lateral to the mid-fusiform sulcus. Follow-up results pending. DISCUSSION/SIGNIFICANCE OF IMPACT: Reduced right FG activation to faces may inform biomarker or response to intervention studies. Mid-fusiform sulcus proved a reliable predictor of functional divides should be investigated on a subject-specific level.

